# Antibacterial and antibiofilm activity of acetone leaf extracts of nine under-investigated south African *Eugenia* and *Syzygium* (Myrtaceae) species and their selectivity indices

**DOI:** 10.1186/s12906-019-2547-z

**Published:** 2019-06-20

**Authors:** Ibukun M. Famuyide, Abimbola O. Aro, Folorunso O. Fasina, Jacobus N. Eloff, Lyndy J. McGaw

**Affiliations:** 1 Phytomedicine Programme, Faculty of Veterinary Science, University of Pretoria, Private Bag X04, Onderstepoort, 0110 South Africa; 2 Department of Veterinary Tropical Diseases, Faculty of Veterinary Science, University of Pretoria, Private Bag X04, Onderstepoort, 0110 South Africa; 3 Emergency Centre for Transboundary Animal Diseases-Food and Agriculture Organization of the United Nations (ECTAD-FAO), House H. Sida, Ali Hassan Mwinyi Road, Ada Estate, Dar es Salaam, Tanzania

**Keywords:** Antibacterial activity, Antibiofilm activity, Cellular safety, Nosocomial bacteria, Myrtaceae, *Syzygium*, *Eugenia*

## Abstract

**Background:**

Antimicrobial resistance (AMR) remains an important global health issue but the gap between AMR and development of new antimicrobials is increasing. Plant extracts may have good activity per se or may be sources of effective antimicrobial compounds which can act against planktonic and/or biofilms of pathogens. We determined the antimicrobial efficacy and cytotoxicity of some under-investigated plants from the Myrtaceae family endemic to South Africa. The ability of the plant extracts to inhibit or destroy pre-formed bacterial biofilms was also determined.

**Methods:**

Based on previous preliminary in vitro screening and on chemotaxonomy, nine species from the Myrtaceae family were selected. The antimicrobial activity of the crude acetone leaf extracts was determined against six common nosocomial pathogens, namely: Gram-positive bacteria (*Bacillus cereus*, *Enterococcus faecalis*, *Staphylococcus aureus*), Gram-negative bacteria (*Escherichia coli*, *Pseudomonas aeruginosa*, *Salmonella* Typhimurium) using a two-fold serial microdilution assay with p-iodonitrotetrazolium violet as growth indicator. The number of antimicrobial compounds present in extracts was determined by bioautography. Cytotoxicity of extracts was determined against Vero kidney cells using a colorimetric tetrazolium-based assay. The total antibacterial activity (TAA) in ml/g and selectivity index (LC_50_/MIC) of the plant extracts were calculated. A modified crystal violet assay was used to determine the antibiofilm activity of the extracts.

**Results:**

*Syzygium legatii*, *Syzygium masukuense*, and *Syzygium* species A had the best activities against Gram-negative and Gram-positive bacteria (MIC) values ranging from 0.04–0.08 mg/ml. *Eugenia erythrophylla* had the best MIC (0.02 mg/ml) against *Bacillus cereus*. Many extracts had relatively low cytotoxicity (LC_50_ > 20 μg/ml) leading to reasonable selectivity indices. Three leaf extracts (*Syzygium masukuense*, *Syzygium* species A, and *Eugenia natalitia*) were moderately cytotoxic (20 μg/ml < LC_50_ < 100 μg/ml). The plant extracts had a good capacity to reduce biofilm formation and good to poor potential to destroy pre-formed biofilms.

**Conclusions:**

The plant species examined in this study had varying degrees of antibacterial activity against bacterial planktonic and biofilm forms with some having good activity against both forms. Several of these selected species may be potential candidates for further investigation to isolate antimicrobial compounds and to determine the mechanism of activity.

## Background

Globally, antibiotics are used to treat infections in humans and animals. In addition to the therapeutic use in animals, antibiotics are commonly added to animal feeds in small quantities as a prophylaxis and for growth promotion purposes [1]. However, there has been a consistent rise in the resistance of microbes to antimicrobials and decreasing ability of the available antimicrobials to treat common infections.

Antimicrobial resistance (AMR) is a major threat to the health and well-being of people and animals with huge impact on food security [2]. However, the world is currently faced with the dilemma of a decline in the number of new therapeutic agents to treat various diseases in both humans and animals [3]. Society may be entering a post-antibiotic era with existing antibiotics gradually becoming ineffective due to resistance. This has major threats to health, as well as national security, for example pandemics and bioterrorism [4]. Current estimates reveal an annual death toll of 700,000 people due to antibiotic resistance and a projection that by 2050, 10 million lives may be at risk if nothing is done to halt the drift towards increasing AMR [5]. Moreover, people living in developing countries where significant morbidity and mortality are due to infectious diseases will be the worst hit by this situation [6].

In 2017, a comprehensive list of priority pathogens was released by the World Health Organization (WHO), including microbes such as *Staphyloccocus aureus*, *Acinetobacter baumannii*, *Streptococcus pneumoniae*, *E. coli*, *Klebsiella* spp., *Enterobacter* spp. etc. [7]. These pathogens have high levels of resistance to most existing antibiotics such as carbapenem, vancomyin, penicillin, ampicillin, and the third-generation antibiotic cephalosporin.

Biofilm formation is one of the resistance strategies of many pathogens which makes them more difficult to treat than their planktonic counterparts [8]. A biofilm is a complex matrix of communities of microorganisms composed of polysaccharides, proteins, and other organic components in which cells bind together to form strong attachments to biotic or abiotic surfaces [9]. Biofilms enable microbes that attach to a surface to persist even in the presence of harsh conditions such as natural host defenses and antimicrobial agents [10]. Therefore, biofilm formation is one of the indirect modes of action by which bacteria are resistant to antibiotics [11] and where they also transfer resistance genes within members of the biofilm micro-community [12]. Many species of bacterial genera of human and veterinary importance such as *Escherichia*, *Staphylococcus*, *Pseudomonas*, *Pasteurella*, *Bacillus*, *Salmonella* etc. cause infections that are difficult to treat due to their ability to form biofilms [13, 14]. Biofilms could be involved in over 60% of microbial infections [15, 16] while two-thirds of all human bacterial infections are caused by the biofilms [17].

The need to tackle these problems coupled with the limited number of new antimicrobials gives enough motivation to increase the search for potential drugs and drug scaffolds from different sources [18]. Efforts focused on natural products research is a promising route of investigation because a significant percentage of new approved antibacterial are either natural products themselves, or natural product derivatives [19].

Plants are a rich reservoir of compounds that have numerous reported biological activities including antimicrobial properties [20–22] thereby becoming a good resource to explore for the discovery of useful and novel antimicrobial products. The World Health Organization also recognizes the place of plants as a mainstay of primary health for over half of the world’s population especially in resource poor countries [23]. Natural products from plants have been recognized as a useful resource that may serve as leads to the discovery of new antimicrobial substances with possible new mechanisms of action. Bioactive plant-based products have the potential to promote the health of animals when included as feed and food components [24].

South Africa is home to a large diversity of plants and many people use plants to meet their health care either due to limited access to Western medicines, particularly in the rural areas, or as adjuncts to conventional medicine even when there is access [6].

We decided to focus our study on plants from the Myrtaceae family because of the promising antimicrobial activities observed in a preliminary evaluation of plants from this family. An extensive study [25] reported that species in the same order and family have a higher chance of yielding interesting antimicrobial compounds. Therefore, selection based on this may be better than from traditional leads alone. Water is the only extractant available to most traditional healers but water does not extract the major bioactive compounds which are usually intermediately polar to non-polar [26, 27].

The Myrtaceae family is globally distributed, consisting of about 145 genera and over 500 known species [28]. Many species belonging to the *Eugenia and Syzygium* genera have been used traditionally to treat different ailments such as diarrhoea, diabetes, reproductive problems, and respiratory conditions [29, 30]. Many studies have reported various pharmacological properties in members of these genera including antimicrobial [31, 32], anti-inflammatory [33], antitumor [34], antioxidant [35] and antidiabetic [36]. Apart from medicinal uses, the plants are commercially explored for their edible fruits, essential oils and for ornamental purposes [31, 37]. There are few or no reports of the biological activity and phytochemistry of the species that we have selected. These plants are largely under-explored for their potential antimicrobial properties. Our research focus is to select plants which may hold potential for development as phytogenic feed additives in combating diarrhoea in livestock management. In this study, we determined the antibacterial activity of acetone crude leaf extracts of nine plant species from the Myrtaceae family native to South Africa against three Gram-positive (*Bacillus cereus*, *Staphylococcus aureus*, *Enterococcus faecalis*) and three Gram-negative (*Escherichia coli*, *Pseudomonas aeruginosa*, *Salmonella* Typhimurium) bacteria. We also determined the cytotoxicity of the extracts since safety is important in consideration for further studies. Furthermore, we investigated the antibiofilm activity of the extracts on the six pathogens to either prevent formation or destroy pre-formed bacterial biofilms because it is an important strategy for microbial persistence in living and non-living tissues.

## Methods

### Collection of plant material, drying and storage

Healthy leaves of nine species from the Myrtaceae family were collected in the summer of 2017, at the Lowveld National Botanical Garden in Nelspruit, Mpumalanga, South Africa. The identities of the plants were verified by Ms. Magda Nel from the Department of Plant and Soil Science, University of Pretoria. Voucher specimens were prepared and deposited in the HGWJ Schweickerdt Herbarium of the University of Pretoria and voucher specimen numbers (PRU) were obtained. The plants selected for this study were: *Eugenia erythrophylla* Strey (PRU 123616), *Eugenia natalitia* Sond. (PRU 123613), *Eugenia woodii* Dummer (PRU 123615), *Eugenia untamvunensis* A.E.van Wyk (PRU 123618), *Eugenia zeyher*i (Harv.) Harv. (PRU 123617), *Syzygium legati*i Burtt Davy & Greenway (PRU 123619), *Syzygium masukuense* subsp. *masukuense* (PRU 123623), *Syzygium* species A (PRU 123622) and *Syzygium gerrardii* (Harv. Ex Hook.f.) Burtt Davy (PRU 123620). *Syzygium* species A could not be identified by the plant taxonomists and may be a new species. Several of these species may be threatened or endangered. Therefore, we did not collect plant material in the veld but only in good botanical gardens with known provenance of the plants. Furthermore, we only investigated leaf material to ensure that it is a sustainable resource.

Methods which were previously developed in the Phytomedicine Programme [38] were used to process the plants. Briefly, leaves were harvested and transported in open mesh loose woven bags into the laboratory. Leaves attacked by microbes or insects were removed and the rest were air-dried indoors at room temperature with good airflow to reduce any microbial attack as well as to facilitate grinding. The dried leaves were then ground to a fine powder using a Janke and Künkel homogenizer Model A10 mill. The leaf powders were weighed and stored in closed glass containers in the dark at room temperature.

### Extraction

Acetone was used as the solvent of choice to extract the powdered leaves as well as to prepare the plant concentrations for the bioassays. Extraction with acetone is considered the best choice because it can extract compounds of a wide range of polarities, it is nontoxic to bioassay systems and easy to remove from extracts [39]. Two grams of ground dry leaf samples were extracted with 20 mL acetone. The mixture was sonicated for 20 min, vigorously shaken, and then poured into a 50 ml polyester centrifuge tube and centrifuged at 4000 x g for 10 min (Hettich Centrifuge, Rotofix 32 A, Labotec, Johannesburg, South Africa). The supernatant was collected and filtered through Whatman No. 1 filter paper into pre-weighed glass vials and concentrated by drying under a stream of cold air. The dried extracts were weighed and the yield obtained by dividing the mass extracted by the initial mass. A concentration of 10 mg/mL (stock solution) in acetone was prepared for use in the assays.

### Analysis of extract by thin layer chromatography (TLC)

Qualitative screening of crude acetone extracts was performed to obtain thin layer chromatography (TLC) fingerprints on aluminum-backed silica gel plates following an established protocol [26]. Three different solvent systems of diverse polarities namely benzene: ethanol: ammonium hydroxide (18,2:0.2, BEA, non-polar basic); chloroform: ethylacetate: formic acid (5,4:1, CEF, intermediate polarity, acidic) and ethylacetate: methanol: water (40,5.4:5, EMW, polar, neutral) were used to elute 100 μg of the extract loaded in a band of 1 cm width on the TLC plates. Visible bands were marked under white light and ultraviolet light (254 nm and 360 nm wavelengths, Camac universal UV light lamp TL-600) and then sprayed with freshly prepared vanillin (0.1 g vanillin, 28 mL methanol, 1 mL sulphuric acid) spray reagent. The plates were then heated to 110 °C until optimal colour development.

### Antibacterial screening

#### Qualitative antibacterial assay by TLC bioautography

To determine the number of antimicrobial compounds in the extracts, bioautograms of the extracts were prepared as described above except that the plates were not sprayed with vanillin. The plates were allowed to dry overnight in a stream of cold air to remove the eluents. The plates were then each sprayed with an actively growing suspension of either *E. coli, S. aureus* or *B. cereus* cultured for 18–24 h at 37 °C until they were wet. The moist plates were then allowed to dry and incubated at 37 °C in a closed plastic humidified sterile container for 24 h to allow the bacteria to grow on the plates. After incubation, the plates were sprayed with 2 mg/mL of freshly prepared p-iodonitrotetrazolium (INT) violet (Sigma) in sterile distilled water and incubated further for 1–2 h for the development of clear zones against a purple-red background which suggests inhibition of bacterial growth by the compounds separated on the chromatograms [40].

#### Quantitative antibacterial assay by minimum inhibitory assay

A widely accepted, simple, reproducible, low cost, and sensitive serial dilution microplate method with INT as indicator of growth [41] was used to determine the minimum inhibitory concentration (MIC) of the crude plant extracts against six bacterial strains in triplicate in three independent experiments. Bacterial cultures were grown overnight in Mueller Hinton broth (Sigma Aldrich, SA) and adjusted to McFarland standard 1 which is equivalent to c. 3.0 × 10^8^ cfu/mL (*Staphylococcus aureus,* ATCC 29213), 2.1 × 10^8^ cfu/mL (*Enterococcus faecalis,* ATCC 29212), 1.3 × 10^8^ cfu/mL (*Bacillus cereus*, ATCC 21366), 3.7 × 10^8^ cfu/mL (*Escherichia coli*, ATCC 25922), 3.5 × 10^8^ cfu/mL (*Salmonella* Typhimurium*,* ATCC 39183) and 3.2 × 10^8^ cfu/mL (*Pseudomonas aeruginosa,* ATCC 27853). The dried extracts were dissolved in acetone to a concentration of 10 mg/mL and 100 μl was added to the first well of a sterile 96-well microtitre plate containing 100 μl of water and serially diluted 1:1 with sterile distilled water. One hundred microliters of appropriately adjusted bacterial cultures were added to each well. The bacteria were subjected to final extract concentrations of 2.5, 1.25, 0.63, 0.32, 0.16, 0.08, 0.04 and 0.02 mg/mL. Gentamicin (Virbac)at the same concentrations as the plant extracts and acetone served as positive and negative controls respectively while water served as sterility control. The bacteria were subjected to decreasing concentrations of acetone starting with 25% in the first well with a two-fold decrease in subsequent wells. It has been established that acetone at these concentrations did not inhibit microbial growth [42]. The microplates were then incubated at 37 °C for 18–24 h. After incubation, the plates were removed from the incubator and 40 μl of 0.2 mg/mL INT dissolved in hot water was added to the wells and incubated further at 37 °C for 2 h. The MIC was determined visually as the lowest concentration that led to growth inhibition [41]. Additionally, the total activity (mL/g) of the extracts was calculated by dividing the mass in mg extracted from 1 g of plant material with the MIC in mg/mL [43]. The total activity incorporates the mass extracted as well as the antibacterial activity and is used to compare the potential use of extracts of different plant species.

### Antibiofilm screening

#### Inhibition of biofilm formation – prevention of initial bacterial cell attachment

The potential of the extracts to prevent initial cell attachment was investigated through the biofilm inhibition assay [44]. Briefly, a 100 μl aliquot of standardised concentration of cultures with OD_560_ = 0.02 (1.0 × 10^6^ CFU/mL) of *Pseudomonas aeruginosa* (ATCC 27853), *Salmonella* Typhimurium (ATCC 39183), *Staphylococcus aureus* (ATCC 29213), *Enterococcus faecalis* (ATCC 29212), *Escherichia coli* (ATCC 25922) or *Bacillus cereus* (ATCC 21366) was added into individual flat-bottomed 96-well microtitre plates and incubated at 37 °C for 4 h without shaking. Then the plates were removed from the incubator and 100 μl (2 mg/ml) aliquots of plant extracts were added in twelve replicates into the wells of 96-well microtitre plates to give a final concentration of 1 mg/mL and then incubated further at 37 °C for 24 h without agitation. Gentamicin (Virbac) served as positive control while acetone and sterile distilled water served as negative controls. The biomass was quantified using the modified crystal violet staining method [45].

#### Inhibition of development of pre-formed biofilms – assessment of destruction of biofilm mass

The ability of the extracts to prevent further biofilm development or destruction of pre-formed biofilms was investigated. A 100 μl aliquot of standardised concentration of cultures with OD_560_ = 0.02 (1.0 × 10^6^ CFU/ml) of *Pseudomonas aeruginosa* (ATCC 27853), *Salmonella* Typhimurium (ATCC 39183), *Staphylococcus aureus* (ATCC 29213), *Enterococcus faecalis* (ATCC 29212), *Escherichia coli* (ATCC 25922) or *Bacillus cereus* (ATCC 21366) was added into individual flat-bottomed 96-well microtitre plates and incubated at 37 °C for 24 h (irreversible attachment phase) or 48 h (mature biofilm) without shaking for the development of a multilayer biofilm. Following respective incubation periods, 100 μl aliquots of plant extracts (2 mg/mL) were added into the wells of a 96-well microtitre plates to give a final concentration of 1 mg/mL and the plates were incubated further at 37 °C for 24 h. Gentamicin (Virbac) at the same concentration as the extracts served as positive control while both acetone and sterile distilled water served as negative controls. The biofilm biomass was assayed using the modified crystal violet (CV) staining assay [45].

### Crystal violet staining assay

The assay was done as previously described [45] with some modifications [44]. Briefly, the 96-well microtitre plates were washed five times with sterile distilled water, air dried and then oven-dried at 60 °C for 45 min. The wells were then stained with 100 μl of 1% crystal violet and incubated at room temperature for 15 min after which the plates were washed thrice with sterile distilled water to remove unabsorbed stain. At this point, biofilms were observed as purple rings at the side of the wells. The semi-quantitative assessment of biofilm formation was done by adding 125 μl of ethanol to destain the wells. A 100 μl aliquot of the destaining solution was transferred to a new sterile plate and the absorbance was measured at 590 nm using a microplate reader (BioTek Synergy). The mean absorbance of the samples was determined, and percentage inhibition of biofilm was determined using the equation below [44]:

$$ \mathrm{Percentage}\ \left(\%\right)\ \mathrm{inhibition}=\frac{{\mathrm{OD}}_{\mathrm{Negative}\ \mathrm{control}}\hbox{-} {\mathrm{OD}}_{\mathrm{Experimental}}\times 100}{{\mathrm{OD}}_{\mathrm{Negative}\ \mathrm{control}}} $$

Although it was not done in the procedure we followed, it would have been interesting to determine if the cells in the biofilm were still viable by using not only the crystal violet assay but also by using e.g. XTT in a different assay. Using this would have enabled us to compare the effect of the plant extracts that generally had an MIC at least ten times lower that the concentration used in the biofilm assay on cells in the biofilm.

### Cytotoxicity

The cellular toxicity of the crude plant extracts to Vero African green monkey kidney cells obtained from the collection of the Department of Veterinary Tropical Diseases, University of Pretoria, was determined using the 3-(4,5-dimethylthiazol)-2,5-diphenyl tetrazolium bromide (MTT) assay [46] with minor modifications [47]. The cells were maintained in minimal essential medium (MEM, Highveld Biological, South Africa) supplemented with 5% foetal calf serum (Adcock-Ingram) and 0.1% gentamicin (Virbac) in a 5% CO_2_ incubator. Cell suspensions were prepared from 70 to 80% confluent monolayer cultures and plated at a density of 5 × 10^4^ cells into each well of sterile flat-bottomed 96-well microtitre cell culture plates. Plates were incubated for 24 h at 37 °C in a 5% CO_2_ incubator before exposure to the extracts. The crude plant extracts were dissolved in acetone (100 mg/mL), and appropriate dilutions were prepared in MEM and added to the wells. Cells were exposed to the various concentrations (0.025 to 1 mg/ml) of plant extracts for 48 h. Doxorubicin (Pfizer) and acetone served as positive and negative controls respectively. After incubation for 48 h, the wells were rinsed twice with 200 μl of phosphate buffered saline (PBS, Sigma) and 200 μl of fresh medium was dispensed into the wells. Then 30 μl (5 mg/ml) of MTT (Sigma) dissolved in PBS was added to each well and the plates were further incubated for 4 h at 37 °C. After this, the medium from the wells was discarded and 50 μl of DMSO was added to the wells to dissolve the formed formazan crystals. Absorbance was measured on a microplate reader (BioTek Synergy) at a wavelength of 570 nm. Each extract concentration was tested in quadruplicate and the assay was repeated three times. The concentration causing 50% inhibition of cell viability (LC_50_) was calculated. Selectivity index (SI) values for the extracts were calculated by dividing cytotoxicity LC_50_ values by the MIC values (LC_50_/MIC).

### Statistical analysis

Experimental results were expressed as mean ± standard error of mean (SEM) of at least three replicates. Where applicable, differences between samples and control were analyzed using one-way analysis of variance (ANOVA) and the significant difference between the means was tested using the Dunnett’s or Tukey’s multiple comparison. Graphpad Instat 6.0 software was used to analyze the data.

## Results

### Plant extracts yield

The yields of the acetone crude leaf extracts of the nine plants is given Table 1. *Eugenia zeyheri* had the highest yield (25.33%) followed by *E. erythrophylla* (18.50%) while *E. umtamvunensis* gave the lowest yield (8.77%).

**Table 1:** Yield of extract, minimum inhibitory concentration (MIC) and total antibacterial activity (TAA) of the nine selected acetone leaf extracts against Gram-positive test bacteria. Values equal to or less than 0.1 mg/ml indicate high activity

		*Enterococcus faecalis*		*Bacillus cereus*		*Staphylococcus aureus*	
Plant	% yield	MIC (mg/ml)	TAA (ml/g)	MIC (mg/ml)	TAA (ml/g)	MIC (mg/ml)	TAA (ml/g)
*Eugenia erythrophylla*	18.50	0.31 ± 0.00	591.05	**0.02 ± 0.00**	9250.00	**0.08 ± 0.00**	2371.79
*Eugenia natalitia*	9.40	**0.08 ± 0.00**	1205.13	**0.08 ± 0.00**	1205.13	0.16 ± 0.00	602.56
*Eugenia woodii*	9.80	0.63 ± 0.00	156.80	0.16 ± 0.00	628.21	**0.08 ± 0.00**	1256.41
*Eugenia umtamvunensis*	8.77	0.16 ± 0.00	561.97	**0.04 ± 0.00**	2247.86	**0.08 ± 0.00**	1123.93
*Eugenia zeyheri*	25.33	1.25 ± 0.00	202.67	**0.08 ± 0.00**	3247.86	**0.08 ± 0.00**	3247.86
*Syzygium legatii*	9.23	**0.08 ± 0.00**	1183.76	**0.03 ± 0.01**	2797.98	**0.08 ± 0.00**	1183.76
*Syzygium masukuense*	10.50	**0.04 ± 0.00**	2692.31	**0.08 ± 0.00**	1346.15	0.16 ± 0.00	673.08
*Syzygium* species A	10.65	**0.04 ± 0.00**	2730.77	0.16 ± 0.00	682.69	**0.04 ± 0.00**	2730.77
*Syzygium gerrardii*	17.00	0.16 ± 0.00	1088.00	**0.04 ± 0.00**	4352.00	**0.08 ± 0.00**	2176.00
Gentamicin	NA	**0.002**	NA	**0.0005**	NA	**0.00013**	NA
Average for extracts	NA	0.3 ± 0.38	NA	0.08 ± 0.05	NA	0.09 ± 0.04	NA

The values in bold indicate good activity

### Bioautography

**Fig. 1:**
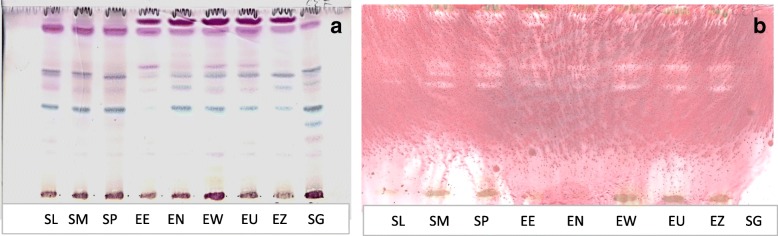
**a** Chromatogram developed in chloroform: ethyl acetate: formic acid (CEF) solvent system of the acetone leaf extracts of the nine plants sprayed with vanillin. **b** Bioautography of *Escherichia coli* developed with CEF; white bands indicate compounds that inhibit the growth of the bacteria. SL = *Syzygium legatii*, SM = *Syzygium masukuense*, SP = *Syzygium* species A, SG = *Syzygium gerrardii*, ER = *Eugenia erythrophylla*, EN = *Eugenia natalitia*, EW = *Eugenia woodii*, EU = *Eugenia umtamvunensis,* EZ = *Eugenia zeyheri*

Of the three solvents used for elution of TLC plates, the intermediate solvent system (CEF) gave the best separation of compounds against *E. coli* (Fig. 1). The retention factor (R_f_) of the active compounds was obtained by dividing the distance moved by the compound by the solvent distance. Four compounds with R_f_ values 0.91, 0.65, 0.59 and 0.56 were observed in *S. masukuense* and *S.* species A while these compounds apart from one compound at R_f_ = 0.59 were present in six of the remaining plants. No clear zone was visible for *S. gerrardii* at the tested concentration indicating that no separated compounds had activity in this assay.

### Antibacterial activity (minimum inhibitory concentration values) and total antibacterial activity

**Fig. 2:**
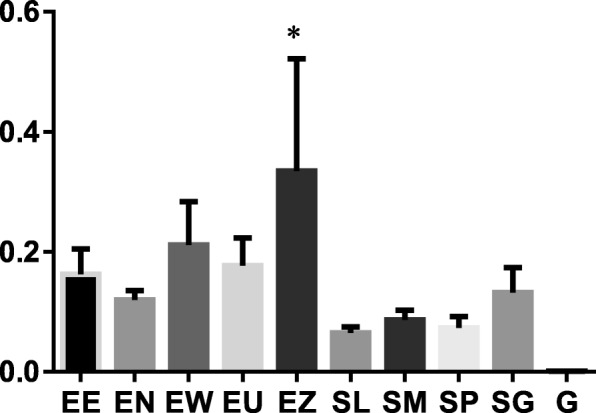
The mean MIC in mg/ml of the acetone leaf extracts of the nine plants against six different bacterial species. EE = *Eugenia erythrophylla*, EN = *Eugenia natalitia*, EW = *Eugenia woodii*, EU = *Eugenia umtamvunensis*, EZ = *Eugenia zeyheri*, SL = *Syzygium legatii*, SM = *Syzygium masukuense*, SP = *Syzygium* sp., SG = *Syzygium gerrardii*, G = Gentamicin (positive control). The negative control (acetone) was higher than 25% (higher than 198 mg/ml). * = shows statistical significant differences (*p* < 0.05) between Gentamicin and *Eugenia zeyheri*

The antibacterial activity of the nine acetone plant extracts in given in Tables 1 and 2. Results showed that the plant extracts were active against the tested bacteria. The MIC values for the extracts ranged between 0.04–0.31 mg/mL for Gram-negative bacteria (Table 2) and 0.02–1.25 mg/mL for Gram-positive bacteria (Table 2). *Bacillus cereus* with a mean MIC of 0.08 (Table 1) was the most susceptible of the Gram-positive bacteria. Of the Gram-negative bacteria, *S.* Typhimurium and *P. aeruginosa*, (mean MICs of 0.14 mg/ml), and *E. coli* (mean MIC = 0.16 mg/mL) were the most susceptible. The total antibacterial activity of each of the plant extract, which was obtained by dividing the quantity extracted from one gram of each plant extract by the MIC value is shown in Tables 1 and 2. *Eugenia erythrophylla*, *S. gerrardii*, *S*. species A, and *E. zeyheri* had the highest mean total antibacterial activities of 2628, 2267, 1939 and 1846 mL/g respectively (Fig. 2).

**Table 2:** Yield of extract, Minimum Inhibitory Concentration (MIC) and total antibacterial activity (TAA) of the nine selected acetone leaf extracts against Gram-negative test bacteria. Values equal to or less than 0.1 mg/ml indicate high activity

		*Escherichia coli*		*Pseudomonas aeruginosa*		*Salmonella* Typhimurium	
Plant	% yield	MIC (mg/ml)	TAA (ml/g)	MIC (mg/ml)	TAA (ml/g)	MIC (mg/ml)	TAA (ml/g)
*Eugenia erythrophylla*	18.50	0.10 ± 0.05	1778.85	0.16 ± 0.00	1185.90	0.31 ± 0.00	591.05
*Eugenia natalitia*	9.40	0.16 ± 0.00	602.56	0.08 ± 0.00	1205.13	0.16 ± 0.00	602.56
*Eugenia woodii*	9.80	0.16 ± 0.00	628.21	0.16 ± 0.00	628.21	0.08 ± 0.00	1256.41
*Eugenia umtamvunensis*	8.77	0.31 ± 0.00	281.15	0.16 ± 0.00	564.10	0.31 ± 0.00	281.15
*Eugenia zeyheri*	25.33	0.13 ± 0.05	1946.15	0.31 ± 0.00	808.31	0.16 ± 0.00	1621.79
*Syzygium legatii*	9.23	0.08 ± 0.00	1179.49	0.08 ± 0.00	1179.49	0.04 ± 0.00	2358.97
*Syzygium masukuense*	10.50	0.08 ± 0.00	1346.15	0.08 ± 0.00	1346.15	0.08 ± 0.00	1346.15
*Syzygium* species A	10.65	0.08 ± 0.00	1371.79	0.08 ± 0.00	1371.79	0.04 ± 0.00	2743.59
*Syzygium gerrardii*	17.00	0.31 ± 0.00	544.00	0.16 ± 0.00	1088.00	0.04 ± 0.00	4352.00
Gentamicin	NA	0.0008	NA	0.0003	NA	0.0002	NA
Average for extracts	NA	0.16 ± 0.09	NA	0.14 ± 0.07	NA	0.13 ± 0.38	NA

### Cytotoxicity

The cytotoxicity activity against Vero cells and their selectivity indices of the nine selected acetone crude extracts is given in Table 3. *Eugenia natalitia* had the highest cytotoxicity (LC_50_ = 38 ± 0.01 μg/mL) followed by *S*. species A (61 ± 0.004 μg/mL), and *S. masukuense* (70 ± 0.01 μg/mL). All the other plant extracts had LC_50_ values greater than 100 μg/mL. The cytotoxicity (mg/mL) and MIC (mg/mL) values are the two variables used to calculate the selectivity index (SI) of a plant extract (SI = LD_50_/MIC), which is a measure of the safety margin of the extract [48]. In this study, *Eugenia zeyheri* had the best average selectivity index of 8.27 against all the pathogens (Table 3) followed by *E. umtamvunensis* (7.90), *E. erythrophylla* (3.53), *S. gerrardii* (2.66) and *S. legatii* (2.44). *E. natalitia* had a poor selectivity index of 0.36.

**Table 3:** Cytotoxicity against Vero cells LC_50_ (μg/ml) and Selectivity index of the nine selected acetone crude extracts. Selectivity index values more than 1 are non-toxic extracts with a relatively good safe margin and promising antibacterial agents

	Selectivity index							
	Cytotoxicity	*E. faecalis*	*B. cereus*	*S. aureus*	*E. coli*	*P. aeruginosa*	*S. typhimurium*	Average
*E. erythrophylla*	250 ± 0.06	0.78	12.55	3.14	2.35	1.57	0.78	3.53
*E. natalitia*	38 ± 0.01	0.48	0.48	0.24	0.24	0.48	0.24	0.36
*E. woodii*	202 ± 0.001	0.32	1.29	2.59	1.29	1.29	2.59	1.56
*E. umtamvunensis*	820 ± 0.05	5.27	21.07	10.54	2.63	5.27	2.63	7.90
*E. zeyheri*	1140 ± 0.17	0.91	14.53	14.53	8.72	3.63	7.27	8.27
*S. legatii*	140 ± 0.05	1.74	4.18	1.74	1.74	1.74	3.48	2.44
*S. masukuense*	70 ± 0.01	1.87	0.94	0.47	0.94	0.94	0.94	1.02
*S.* species A	61 ± 0.004	1.57	0.39	1.57	0.79	0.79	1.57	1.11
*S. gerrardii*	200 ± 0.03	5.11	1.28	2.56	0.64	1.28	5.11	2.66
Doxorubicin	12.07 ± 0.41	ND	ND	ND	ND	ND	ND	ND

### Prevention of cell attachment: antibiofilm activity/ anti-adhesion

The effect of acetone crude extracts on the attachment and inhibition of biofilm formation is given in Table 4. According to established criteria [44], percentage inhibition values between 0 to 100% signify inhibition of biofilm, while enhancement of growth is reflected by values below 0%. Above the 50% inhibition mark the activity is regarded as good, while it is poor if it is between 0 and 49%.

**Table 4:** Effect of acetone crude leaf extracts of nine plants against biofilm formation by Gram-negative and Gram-positive bacteria. Inhibition of biofilm formed is expressed as percentage (**%**) biofilm inhibition. The numbers > 0% ≥ 50% show low activity, > 50% (in bold) show high activity against the bacteria. Negative values denote enhancement of biofilm [44]

	*E. coli*	*P. aeruginosa*	*S.* Typhimurium	*E. faecalis*	*B. cereus*	*S. aureus*
*E. erythrophylla*	**83**	**76**	**88**	41	**80**	**83**
*E. natalitia*	**77**	**64**	**68**	47	41	35
*E. woodii*	−10	**58**	49	−19	**71**	42
*E. umtamvunensis*	27	**83**	**105**	**57**	**77**	**73**
*E. zeyheri*	34	**65**	22	−104	**74**	**83**
*S. legatii*	**100**	**59**	**100**	**71**	**85**	**86**
*S. masukuense*	34	−62	**84**	**125**	44	− 155
*S*. species A	**81**	− 276	**82**	−96	**97**	−29
*S. gerrardii*	−15	−125	**122**	**114**	**146**	**68**
Gentamicin	**83**	**75**	**81**	**88**	**84**	**96**

The plant extracts had varying degrees of activity on the prevention of attachment. Four of the extracts had good prevention of biofilm attachment against *E. coli* (> 50%), three extracts had inhibition less than 50% while the extracts of *E. woodii* and *S. gerrardii* enhanced the biofilm growth and hence were not included in the biofilm development assay.

All plant extracts from the *Eugenia* species prevented attachment of *P. aeruginosa* with values above 50%. Apart from *S. legatii*, no *Syzygium* species inhibited cell attachment but rather enhanced biofilm formation based on the observed negative percentage inhibition values.

All the extracts, at inhibition > 0%, prevented attachment of *S.* Typhimurium although the activity of *E. woodii* and *E. zeyheri* was below 50%*.*

Six out of the nine plant extracts evaluated against *E. faecalis* prevented cell attachment while *E. woodii*, *E. zeyheri*, and *S.* species A enhanced biofilm attachment and growth.

In this study, all the plant extracts prevented the attachment of *B. cereus* although *E. natalitia* and *S. masukuense* had values lower than 50% (Table 4).

Of the nine extracts tested against *S. aureus*, only *S. masukuense* and *S.* species A extracts did not prevent the attachment of the bacteria although these two plants had good MICs (Table 1). *Eugenia natalitia* and *E. woodii* extracts, however had poor activity with values between 0 and 50%.

### Inhibition of development of pre-formed biofilms – assessment of destruction of biofilm mass

The activities of plant extracts with good anti-cell attachment properties on the prevention or reduction of further biofilm development in 24 and 48 h preformed biofilms are presented in Table 5.

**Table 5:** Effect of acetone crude leaf extracts of nine plants against Gram-negative and Gram-positive bacteria after 24 and 48 h pre-formed biofilm expressed as percentage (**%**) biofilm inhibition. The numbers > 0% ≤ 50% indicates low activity, > 50% (in bold) indicates high activity against the bacteria. Negative values denote enhancement of biofilm [44]

	*E. coli*		*P. aeruginosa*		*S.* Typhimurium		*E. faecalis*		*B. cereus*		*S. aureus*	
	24 h	48 h	24 h	48 h	24 h	48 h	24 h	48 h	24 h	48 h	24 h	48 h
*E. erythrophylla*	− 150	−26	− 261	− 364	− 275	30	−21	−67	−254	− 194	5	− 115
*E. natalitia*	**82**	− 142	− 362	− 178	**73**	21	−211	− 7	− 35	− 44	− 248	43
*E. woodii*	ND	ND	− 218	− 16	29	− 175	ND	ND	−179	− 171	− 147	− 488
*E. umtamvunensis*	43	28	− 126	−140	33	**94**	**86**	−155	−12	−7	**75**	38
*E. zeyheri*	**237**	−4	− 263	− 65	**341**	**66**	ND	ND	− 260	−47	− 299	− 326
*S. legatii*	−115	− 146	− 85	− 82	−143	− 273	39	4	−275	− 216	**56**	− 41
*S. masukuense*	**218**	**95**	ND	ND	**154**	**162**	**138**	− 426	9	**257**	ND	ND
*S.* species A	38	**142**	ND	ND	43	**151**	ND	ND	− 192	48	ND	ND
*S. gerrardii*	ND	ND	ND	ND	**59**	−142	**71**	**69**	**96**	−103	**142**	**133**
Gentamicin	**90**	**64**	**78**	**85**	18	**103**	**81**	**72**	27	12	**75**	**53**

*ND* Not determined

Of the seven plant extracts evaluated, five plant extracts reduced *E. coli* biofilm biomass at 24 h post-development (Table 5). Out of these five, three (*E. natalitia*, *E. zeyheri* and *S. masukuense*) had percentage inhibition values above 50%. After 48 h pre-formed biofilm, *E. umtamvunensis* extracts had poor antibiofilm activity (28%) while *S. masukuense* and *S.* species A prevented biofilm development by over 50% while the remaining extracts showed enhancement of biofilm. None of the plant extracts was able to destroy the biofilm of *P. aeruginosa*.

Antibiofilm activity against *S.* Typhimurium showed that after a 24 h pre-formed biofilm, *E. woodii, E. umtamvunensis* and *S.* species A had poor biofilm inhibiting activity of 29, 33, and 43% respectively while *E. natalitia, E. zeyheri, S. masukuense,* and *S. gerrardii* had good (> 50%) antibiofilm activity. *Eugenia erythrophylla* and *S. legatii* enhanced biofilm formation at this period. In contrast, *E. umtamvunensis*, *E. zeyheri, S. masukuense* and *S.* species A had good antibiofilm activity after a 48 h pre-formed biofilm.

Of the six plants investigated, four reduced the 24 h *E. faecalis* biofilms and out of these, only *S. gerrardii* was active against the 48 h pre-formed biofilm (Table 5).

In the activity against *B. cereus* (Table 5), of all the investigated plants, *S. masukuense* had some activity, albeit poor, against the 24-h biofilm but had improved activity in the 48 h pre-formed biofilm. Also, *S.* species A enhanced of biofilm formation at 24 h but had some inhibition against the 48-h biofilm. *Syzygium gerrardii* was only effective in reducing the 24 h biofilm but was not active against the 48-h biofilm. Like *P. aeruginosa*, most of the plant extract did not have any effect on *B. cereus* biofilms at 24 h and 48 h.

The extracts of *E. umtamvunensis*, *S. legatii* and *S. gerrardii* had inhibitions of above 50% against 24 h *S. aureus* biofilm, *E. erythrophylla* had poor activity while others showed biofilm enhancement. *Syzygium gerrardii* had a good activity against *S. aureus* 48 h biofilm.

## Discussion

The yield of a plant extract is important in calculating the total activity to compare plants for bioprospecting [43]. Bioautography was used to separate the antibacterial compound(s) present in plant extracts to provide more information on the diversity of the antibacterial compounds in the different plant species extracts. Active compounds in the plant extracts were mostly present in the intermediate polarity extracts. The portion of the chromatogram that shows white zones surrounded by pinkish areas denotes the Rf value of the active compound in each extract (Fig. 1b). In most of the extracts, more than one active compound was observed.

The minimum inhibitory concentration of the plant extracts was determined. The lower the MIC is the better is the activity [49]. Antimicrobial activity of plant extracts have been classified as good (MIC< 0.1 mg/mL), moderate (0.1 ≤ MIC ≤0.625 mg/mL) and weak (MIC > 0.625 mg/mL) [50]. Most of the extracts had good activity against at least two of the bacteria examined.

The Gram-negative bacteria generally had a higher resistance to the plant extracts than Gram-positive bacteria [51, 52]. This may be attributed to the distinct feature of the morphology of cell walls of Gram-negative bacteria which, in contrast to those of Gram-positive bacteria, comprise a hydrophilic lipopolysaccharide outer layer highly resistant to the penetration of antibacterial agents, as well as the presence of some enzymes in the periplasmic space which break down antibacterial molecules [53].

**Fig. 3:**
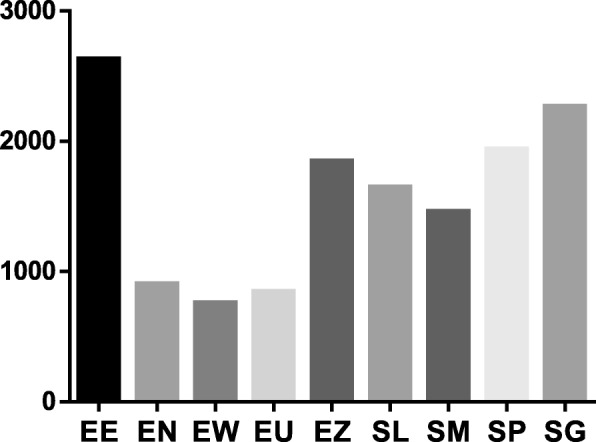
The mean MIC in mg/ml of the acetone leaf extracts of the nine plants against six different bacterial species. EE = *Eugenia erythrophylla*, EN = *Eugenia natalitia*, EW = *Eugenia woodii*, EU = *Eugenia umtamvunensis*, EZ = *Eugenia zeyheri*, SL = *Syzygium legatii*, SM = *Syzygium masukuense*, SP = *Syzygium* sp., SG = *Syzygium gerrardii*, G = Gentamicin (positive control). The negative control (acetone) was higher than 25% (higher than 198 mg/ml). * = shows statistical significant differences (*p* < 0.05) between Gentamicin and *Eugenia zeyheri*

More plant extracts from the *Syzygium* genus had higher activity against the Gram-negative species than those from the *Eugenia* genus (Table 2). Of the nine plant species, *S. legatii* had a broad spectrum of antibacterial activity with good activity on all the bacteria tested. With the exception of the *E. zeyheri* extracts, there were no statistically significant differences (*P* > 0.05) between the activity of the other extracts and gentamicin (Fig. 3).

The potency of a plant extract, referred to as the total antibacterial activity (TAA) can be determined on the basis of both its MIC in mg/mL and extract yield in mg/g [49]. This value gives the volume (ml) to which the extract obtained from one g of plant material can be diluted and still be able to inhibit the bacteria. In this study, *E. zeyheri* had the best mean TAA of 2628 mL/g due to the large extract yield. This means that if 2.628 L of the solvent is added to the quantity extracted from 1 g of the plant, bacterial growth can still be inhibited. The TAA is useful to determine the most suitable plant extract for compound isolation and bioprospecting [26].

Although it is often wrongly assumed that plant extracts and other natural products are safe, it is necessary to determine their cytotoxicity to provide scientific evidence whether they are safe or not [48]. A plant extract is considered to be highly cytotoxic when the LC_50_ is 20 μg/mL and below [54]. It should however be borne in mind that in vitro cellular toxicity may not equate to whole animal toxicity because different factors such as gut interactions and bioavailability play a role. Therefore, acute and chronic animal toxicity is needed to determine the toxicity of the extracts [55]. All of the plant extracts investigated had relatively low cytotoxicity with LD_50_ values higher than the predetermined cut-off point (20 μg/mL) (Table 3).

It is important to consider the selectivity index (SI) of a plant extract. A SI value greater than 1 means that the extract is more toxic to the pathogen than to normal body cells. The higher the SI value, the safer is the plant extract and the higher the potential to be developed as a safe herbal product. The extracts of many of the plants investigated in this study have potential to be developed into useful products to control antimicrobial infections by herbal remedies. Alternatively, isolation of active compounds can form a template for the development of new drugs.

Bacterial biofilm remains a global threat to health due to high refractoriness to treatment and the ability to aggravate nosocomial infections. Hence, search for novel efficacious molecules to tackle this problem is a priority [56]. In this study, the activities of the plant extracts were tested against the biofilms of the bacteria species. This appears to be the first study to determine the antibiofilm activity of the selected plant extracts. The ability of antibacterial agents to inhibit formation of or destruction of biofilms hold promise for reducing colonization of surfaces and epithelial mucosa by microbes [57].

With the exception of *E. woodii* and *S. gerrardii* extracts, all the other extracts prevented the formation of *E. coli* biofilms. All the plant extracts prevented *S. Typhimurium* and *B. cereus* biofilms. *Syzygium legatii*, *S. masukuense*, and *S.* species A had an excellent MIC of 0.08 mg/mL against planktonic *P. aeruginosa.* With the exception of *S. legatii* no *Syzygium* species was able to prevent biofilm production in *P. aeruginosa*. All the *Eugenia* species in this study inhibited attachment of *P. aeruginosa* by over 50% indicating a good anti-attachment property. The attachment of *S. aureus* and *E. faecalis* was also inhibited by most of the plant extracts.

The excellent ability of the plant extracts to interfere with the initial stage of biofilm formation of the six bacterial isolates may be attributed to interference with forces (such as Brownian, sedimentation, Lifshitz–Van der Waals and electrostatic interaction forces) that favour the deposition and adherence of bacteria to surfaces [58]. Also, since certain organic and inorganic molecules and other nutrients are important for cell growth and hence cell adhesion [59], it is possible that the plant extracts may inhibit the availability of nutrients. The active plant extracts may hold promise for reduction of colonization surfaces and various epithelial of the body, thereby preventing infections. A similar observation was made in a previous study on the ability of acetone and ethanol crude extracts of *Psidium guajava* (Myrtaceae) to prevent attachment of *Streptococcus mutans*, a known biofilm former on oral surfaces [60]. In another study, leaf extracts of *E. leitonii*, *E. brasiliensis*, *E. myrcianthes* and *E. involucrate* had good antiadhesive activity against *Candida albicans* [31]. This shows that *Eugenia* and *Syzygium* species possess compounds with promising antibiofilm activity.

This study assessed the ability of plant extracts to destroy or prevent further formation of established biofilms at 24 h and 48 h. Only plant extracts with anti-attachment activity was included in this study.

The plant extract of *S. masukuense* had excellent activity against the 24 h and 48 h *E. coli* biofilms. Compounds from this plant extract may be useful to combat diseases such as otitis media and urinary tract infections caused by *E. coli* is biofilms, at acute or chronic stages [61].

The anti-*Salmonella* activity shown by the plants even after 48 h biofilm highlights their potential for control of *Salmonella* biofilms. In a similar study [62], natural compounds such as eugenol, thymol and carvacol inhibited *S.* Typhimurium biofilm formations by more than 50%. Possible applications may be spraying of vegetables by natural compounds for decontamination of food pathogens such as *Salmonella* [63].

*Enterococcus faecalis* is a common biofilm former usually implicated in urinary tract infections, wound infections, and dental infections [64]. The extracts of *Eugenia umtamvunensis* and *S. masukuense* had good activity against the 24 h *E. faecalis* biofilm while *S. gerrardii* had excellent activity against both 24 h and 48 h biofilms. There is a potential to develop herbal products from these plant extracts as mouth rinses for oral care, as well as for topical applications to treat wounds in medical and veterinary care. It may be possible to isolate an active compound against *E. faecalis* biofilm from *S. gerrardii*.

*Syzygium gerrardii* had the best activity against 24 h and 48 h *S. aureus* biofilms. In a related study, the essential oil from *Syzygium aromaticum* had good activity against *Staphylococci* biofilms associated with mastitis in the dairy industry [65]. *Syzygium gerrardii* may be a good candidate for the development of anti-staphylococcal products that may find application as sanitizers to clean surfaces. Also, it may contain active compounds that may form template for the development of potent drugs to treat staphylococcal infections in humans and animals.

The biofilm of *P. aeruginosa* was the most difficult to eradicate among all the tested bacteria. Although some of the extracts had a good potential to prevent attachment, none was able to get rid of pre-formed biofilms after 24 and 48 h. In contrast, most of the extracts had good minimum inhibitory activity against *P. aeruginosa* in the quantitative minimum inhibitory assay. *Pseudomonas aeruginosa* is a notorious biofilm producer and its biofilms cause the highest number of acute and chronic infections, especially in the excretory and respiratory systems [66, 67], and it can also colonize medical devices and body implants [12]. The enhancement of biofilm production by some of the extracts confirm initial reports that some natural compounds may promote the growth of microbes [68].

Compounds present in the plant extracts may be responsible for the various antibiofilm activities observed in this study. In a recent review, isolated natural compounds from plants had antibiofilm activity against different bacterial pathogens [69]. In the future, attempts will be made to isolate compounds which may responsible for the antibiofilm effects of the active plant extracts.

Generally, it was more difficult to eradicate pre-existing biofilms by the extracts in this study. Some reports have also noted that it is less difficult to inhibit cell attachment than to get rid of established biofilm [70, 71]. This confirms that pathogens are able to resist the action of antimicrobials more when they exist in biofilms and their infections are able to persist on different biotic and abiotic surfaces [61]. Factors which cause resistance in biofilms include presence of an extracellular polymetric matrix which causes strong attachment of microbes to surfaces and low antibiotic penetration or increased activity of efflux pumps which expel antimicrobial agents from cells [10]. The plant extracts may have interfered with any of these factors. The plant extracts may have also interfered with the cell to cell communication strategies (quorum sensing) of the bacteria, thereby reducing biofilm formation [72].

The activity of the plant extracts against biofilms at different stages of development highlights their potential usefulness in clinical applications. Such applications may help to enhance the immunological defense of infected hosts against bacterial cell populations, especially those in biofilms, and subsequent host clearance and reduction of disease symptoms [66]. Some of the active extracts may potentially be developed into herbal products which can be useful in alleviating diarrhoeal symptoms and enhancing productivity when added to livestock feed as replacement to antibiotics. The use of antibiotics as additives to livestock feeds has been banned in many countries worldwide due to issues of transfer of antimicrobial resistance to humans [73]. More studies are needed to elucidate the mechanism of antibiofilm action of the active plant extracts in this study.

## Conclusions

Little was known about the antimicrobial activities of the selected plants in this study. Our results showed that the crude extracts of the plants had good activity on the planktonic and sessile forms of the bacterial species investigated. Many of the plants had low toxicity which makes them good candidates for possible development into herbal products or for isolation of novel pure compounds that can serve as templates for new antimicrobial drugs. The crude extracts of some of the plants have potential as phytogenic feed additives that can replace in-feed single compound antibiotics commonly added to livestock feeds as growth promoters, especially when animal feed trials are performed. Although we used bacterial strains recommended by the Clinical and Laboratory Standards Institute to compare activity of different antibiotic substances, it will be interesting to determine the activities of these plants against clinical bacterial isolates to further assess their relevance for use in clinical conditions. It is imperative to determine the mode of antibacterial action of the extracts and how they act on the different pathogens. To a reasonable extent, our findings have provided credence to the value of selecting plants for antimicrobial investigation on a taxonomic basis [25]. These results may stimulate further biological research on these and other under-explored plant species in the Myrtaceae family, especially those native to southern Africa.

## Data Availability

The datasets used and/or analyzed during the current study are available from the corresponding author on reasonable request.

## Abbreviations

ATCC: American Type Culture Collection
CFU: Colony forming unit
DMSO: Dimethyl sulphoxide
INT: ρ-iodonitrotetrazolium violet
LC^50^: Lethal concentration (50%)
MIC: Minimum inhibitory concentration
MTT: 3-(4,5-dimethylthiazol-2-yl)-2, 5-diphenyltetrazolium bromide
OD: Optical density
PBS: Phosphate buffer saline
SD: Standard deviation
SI: Selectivity index
TAA: Total antibacterial activity
